# I Will Do It If I Enjoy It! The Moderating Effect of Seeking Sensory Pleasure When Exposed to Participatory CSR Campaigns

**DOI:** 10.3389/fpsyg.2015.01940

**Published:** 2016-01-05

**Authors:** Salvador Ruiz de Maya, Rafaela Lardín-Zambudio, Inés López-López

**Affiliations:** Marketing Department, University of MurciaMurcia, Spain

**Keywords:** CSR associations, participation, sensory pleasure, skepticism, attitude

## Abstract

In an attempt to gain differentiation, companies are allocating resources to corporate social responsibility (CSR) initiatives. At the same time, they are giving consumers a more active role in the process of creating value. In this sense, consumer participation represents a new approach to gain competitive advantage. However, the effectiveness of consumer participation in CSR campaigns still remains unknown. With the purpose of shedding light on this issue, this paper shows that participatory CSR campaigns lead to greater consumer perceptions of CSR, which in turn results in more favorable attitudes toward the company. Furthermore, the effect is stronger for sensory pleasure seekers, whose involvement with the experience is greater. The findings contribute to the CSR literature and reveal important implications for marketers.

## Introduction

In the current marketplace, companies must attain differentiation and credibility to develop strong and long-term relationships with consumers. To achieve these goals, a growing number of firms are allocating resources to corporate social responsibility (CSR; Lee et al., 2012).

Both the way consumers perceive the information on CSR and the level of stimulation this information generates influence attitudes and behaviors (Brown and Dacin, 1997; Sen and Bhattacharya, 2001). For example, inferences drawn from a company's prosocial actions can change even product evaluations (products are perceived as performing better), regardless of whether consumers are observing or experiencing the product (Chernev and Blair, 2015). A large body of research has empirically established that consumers' perceptions of firms' motives for engaging in CSR influence their evaluations of and responsiveness to CSR (Ellen et al., 2006). In general, consumers are aware that CSR can contribute to company image formation, and thus their interests in CSR activities continue to rise (Lee et al., 2012; Schmeltz, 2012). However, to some extent current approaches to CSR are still disconnected from companies' global strategy, thus masking their opportunities to benefit society (Porter and Kramer, 2006). This flaw highlights the need to shed light on the connection between CSR actions and other mechanisms in order to assist consumer persuasion.

In the past two decades, consumers have begun taking more active roles in companies' efforts to compete for and create value (Prahalad and Ramaswamy, 2000). That is, consumers are no longer passive audiences but active coproducers of value (Dong et al., 2008). Bendapudi and Leone (2003) linked high levels of consumer participation to competitive effectiveness. In support of this, extant literature in marketing has found that consumer participation has a positive effect on consumer behavior (Dellande et al., 2004; Chan et al., 2010). However, while the impact of CSR and participation on consumer behavior has been widely demonstrated in the literature, whether consumer participation in CSR activities increases the effectiveness of the latter still remains unexplored. In addition, the notion that consumers seek out pleasurable products and experiences (Hirschman and Holbrook, 1982) must be taking into account. Because participation may be associated with use of a product or going through an experience, the possibility of experiencing sensory pleasure may influence consumers' perceptions of the CSR activities in which they participate.

With the aim of shedding light on this issue, the goal of this paper is 2-fold. First, we aim to demonstrate that the participatory nature of CSR campaigns influences consumer perceptions. Second, we assess whether the dispositional trait of sensory pleasure seeking moderates this effect. The structure of the paper is as follows: We begin with a review of the relevant literature and present the theoretical background. Then, we develop a set of hypotheses and describe the method. Finally, we report the main results and discuss conclusions.

## Conceptual framework and hypotheses

In the past decade, researchers have shown interest in understanding how CSR activities influence consumer behavior (Marin and Ruiz, 2007; Boulouta and Pitelis, 2014). By engaging in CSR and signaling this engagement to consumers, companies can improve consumer-related outcomes (Luo and Bhattacharya, 2006). Companies can use CSR as an instrument to enhance firm image through its effects on consumers' intentions and attitudes (Brown and Dacin, 1997; Sen and Bhattacharya, 2001; Bhattacharya and Sen, 2004). That is, CSR initiatives can be central, distinctive, and enduring, thus contributing to more positive consumer evaluations of the company (Marin et al., 2009).

Consumers attribute many corporate motives to CSR engagement related mainly to company contributions to society (Ellen et al., 2006). Attribution theory states that people attribute causes to events and that their cognitive perceptions influence their subsequent attitudes and behavior (Kelley and Michela, 1980). In addition, and according to the persuasion knowledge model (Friestad and Wright, 1994), consumers accumulate knowledge on persuasive motives and tactics (Groza et al., 2011) and then use such knowledge to make inferences about firm ultimate motives. Thus, what consumers know about a company influences their associations. CSR associations reflect an organization's status and activities with respect to its perceived obligations to society and can exert different effects on consumer responses (Brown and Dacin, 1997). In summary, consumers' associations with CSR activities influence their evaluations of and responsiveness to CSR (Becker-Olsen et al., 2006; Ellen et al., 2006). Using the persuasion knowledge model and attribution theory as theoretical foundations, we posit that a CSR campaign is a persuasive attempt to create positive consumer perceptions.

### Consumer participation

Being consumer oriented is not enough for firms to successfully compete in today's marketplaces. Firms must learn from and collaborate with consumers to create value that meets their individual and dynamic needs (Prahalad and Ramaswamy, 2000). Ulrich (1989) argues that involving consumers is a powerful way to increase consumer loyalty and commitment. The service literature lends further support to this claim, finding a positive and significant relationship between consumer participation and commitment (Bettencourt, 1997).

Previous research claims that as consumers' involvement with a firm increases, the company gains more opportunity to shape consumer perceptions (Bowen, 1986). Thus, consumers with high levels of involvement may have perceptions of quality and levels of satisfaction that differ from those who are less involved (Kelley et al., 1990). In line with this, research in marketing has underscored the importance of consumer participation (Bettencourt, 1997), or “the degree to which the consumer is involved in producing and delivering the service” (Dabholkar, 1990, p. 484). Participation can include tasks such as spending time interacting, responding to questions, or providing information on product specifications, brand preferences, and price range (Dabholkar and Sheng, 2012).

The potential of consumer participation has attracted research attention because of the assumption that when consumers participate actively, organizations can gain competitive advantage through greater sales volume, enhanced operating efficiencies, positive word of mouth, reduced marketing expenses, and enhanced consumer loyalty (Reichheld and Sasser, 1990). One stream of research focuses on the reasons consumers should engage in the service provision process and deals with the economic benefits of consumer participation (Bendapudi and Leone, 2003). A second stream also considers consumer motivations to cocreate a service, analyzing the motivation of self-service consumers (Bateson, 1985) and exploring key factors that influence initial trial decisions, consumer traits, and situational factors on technology adoption (Meuter et al., 2005). A third stream focuses on managing consumers as partial employees (Bendapudi and Leone, 2003), assuming that consumers' active participation in service provision leads to greater perceived service quality and enhanced consumer satisfaction (Dabholkar, 1990; Claycomb et al., 2001).

In addition, extant literature in marketing has found that consumer participation has a positive effect on consumer behavior (Dellande et al., 2004; Chan et al., 2010). Thus, research has shown the positive effect of participation in the areas of consumer decision making (Cermak et al., 1994; Matzler et al., 2005), brand loyalty (Bagozzi and Dholakia, 2006), commitment to the brand (Casaló et al., 2007), quality perceptions (Dabholkar, 1990), word of mouth (Kim and Jung, 2007), trust (Ouschan et al., 2006), affective commitment to the product (Atakan et al., 2014) and sensory perceptions (Troye and Supphellen, 2012). Bendapudi and Leone (2003) show that when the service outcome is better than expected, participating consumers are more satisfied than non-participating consumers. Matzler et al. (2005) report that in contexts characterized by high consumer participation, consumer satisfaction and other postpurchase responses (e.g., positive word of mouth, loyalty) are more favorable. Additionally, participation has been related to higher employees' satisfaction and performance (Yi et al., 2011). Therefore, as consumers' participation increases, subsequent outcomes become more positive. Encouraging consumer participation, then, may represent a good opportunity to gain competitive effectiveness and should deliver value to both customers and firms (Bendapudi and Leone, 2003).

### Consumer participation and CSR associations

Prior research has shown that firms can generate more favorable attitudinal responses from consumers when they are proactively engaged in CSR activities rather than acting reactively (Becker-Olsen et al., 2006; Wagner et al., 2009). This effect finds support in the employee participation literature, which shows that participation influences perceptions of, for example, service quality (Dabholkar, 1990). In the same vein, Bowen (1986) suggests that as consumers increase their level of involvement with a firm, the firm gains the opportunity to shape their perceptions, and Kelley et al. (1990) report that consumers with high levels of service involvement have perceptions of service quality and levels of satisfaction that differ from consumers not highly involved in the participatory role. Furthermore, Claycomb et al. (2001) demonstrate that consumer participation results in more positive perceptions of the organization and that higher levels of consumer participation in the service delivery process are associated with positive perceptions of service encounter performance. In this context, consumer participation in a CSR campaign reflects the degree to which the consumer is involved in CSR activities.

The findings on consumer participation related to a company's main activity can also apply to other activities developed by the company, such as those related to CSR. Therefore, we propose that the participatory nature of the CSR campaign will have a positive effect on consumer perceptions of CSR. We contend that consumers' participation in CSR activities will result in greater involvement, greater understanding, and deeper knowledge, which in turn will lead to perceptions of more CSR effort and, therefore, greater CSR associations (Stanaland et al., 2011). CSR associations influenced by corporate efforts depend to some degree on effective firm communication with external audiences and represent consumers' perceptions. Therefore, we propose the following:

*H1:* Consumers exposed to a participatory CSR campaign will have greater CSR associations than consumers exposed to a non-participatory campaign.

### Motivation for sensory pleasure and CSR associations

Prior research has documented that consumers seek out pleasurable products and experiences (Hirschman and Holbrook, 1982) and show motivational differences in pursuing favorable experiences and avoiding unpleasant ones (Chapman and Chapman, 1985). The motive for sensory pleasure (MSP) describes the individual drive to seek out pleasant auditory, visual, tactile, olfactory, and taste experiences and to similarly avoid unpleasant sensory experiences (Eisenberger et al., 2010). Recently, Eisenberger et al. (2010) noted that high MSP individuals engage in greater pursuit of favorable experiences.

Moreover, personality theorists have examined dispositional differences in the enjoyment of sensory experiences (Chapman and Chapman, 1985). Thus, some individuals are high sensory pleasure seekers and others are less biased in relation to this pursuit of pleasure. According to Jackson (1984, p. 7), the highly sentient person “notices smells, sounds, sights, tastes, and the way things feel; remembers these sensations and believes they are an important part of life; is sensitive to many forms of experience; [and] may maintain an essentially hedonistic or aesthetic view of life.” As a result, consumers can serve as “moderators” of pleasure through their idiosyncratic reactions to product experiences (Alba and Williams, 2013).

Prior research has shown the importance of pleasure in consumer behavior, demonstrating that emotional states (pleasure and arousal) are important determinants of purchase behavior (Sherman et al., 1997; López López and Ruiz de Maya, 2012). Fiore (2002) finds that sensory pleasure from a catalog page positively affected approach responses of global attitude. In addition, theoretical support exists for the link between pleasure and satisfaction. As Bigné et al. (2005) note, consumers who derive pleasure from an experience are more likely to exhibit positive behavioral intentions, such as positive word of mouth, satisfaction, and intention to return to the store.

However, motivation for sensory pleasure may work in the opposite direction. If consumers motivated for sensory pleasure do not experience what they are looking for (sensory pleasure), their interest in the stimulus may be low, which will imply a lower processing too (Petty and Cacioppo, 1986). In addition, while motivation for sensory pleasure is clearly related to emotional involvement (Nurse et al., 2010), Eisenberger et al. (2010) point out to the uniqueness of this personality trait as separated from need for cognition (Cacioppo et al., 1996) and, as such, subjects highly motivated to seek for sensory pleasure will base their behavior on emotions associated to the activity rather than cognitions (that require processing) related to how good the company is doing with they CSR activity it is developing. The application of this reasoning to CSR activities, therefore, leads us to propose that those who are highly motivated to seek sensory pleasure will process much less the campaign and will show less CSR associations than those who are less motivated to search for sensory pleasure. Formally,

*H2:* The higher the consumers' motivation for sensory pleasure, the lesser their CSR associations will be when exposed to a CSR campaign.

### The moderating role of motivation for sensory pleasure

Personal relevance theory holds that individuals have a level of interest in and give particular importance to a cause (Antil, 1984). Therefore, when a cause is important to consumers, they will feel more interested and involved in the action. Previous research has shown that involvement significantly moderates how stimulus cues influence brand evaluation and communication effectiveness (Maoz and Tybout, 2002). More important, involvement is positively related to information processing (Leigh and Menon, 1987). Therefore, because CSR campaigns influence consumers' cognitive responses, those more involved will process the information of the CSR campaign more thoroughly and will value the social nature of the campaign more than those less involved (Gupta and Pirsch, 2006).

Consumer participation is a behavior that reflects a state of involvement (Cermak et al., 1994) that can be increased by other sources of motivation. As Eisenberger et al. (2010) argue, “high MSP individuals' enhanced motivation [will] produce greater pursuit of favorable nature experience.” Accordingly, the nature of the campaign (participatory or non-participatory) should generate different responses, depending on the participants' additional involvement (i.e., the level of consumers' motivation to seek sensory pleasure). From these arguments, we propose that in a participatory campaign, consumers who are sensory pleasure seekers will be more involved with the campaign and, consequently, will have more CSR associations (perceive greater CSR). Therefore, we propose the following:

*H3*: When exposed to a participatory CSR campaign, the effect on CSR associations will be stronger for consumers with high motivation for sensory pleasure than for those with low motivation for sensory pleasure.

### Consumer skepticism of CSR associations

Skepticism refers to a person's tendency to doubt, disbelieve, and question (Forehand and Grier, 2003). Research in the field of economics and business views skepticism as a potential consumer response to the actions of companies (Skarmeas and Leonidou, 2013) and defines it as consumers' distrust of or disbelief in companies (Webb and Mohr, 1998). The limited research on this topic notes that skepticism toward a company (negative assessment) occurs when consumers attribute selfish motives to the company actions (Webb and Mohr, 1998; Ellen et al., 2006). Thus, skepticism predisposes consumers to doubt the veracity of the communication activities of the company (Obermiller and Spangenberg, 1998). Indeed, consumers show a natural tendency to be skeptical of advertising (Obermiller and Spangenberg, 1998), though the extent to which they are skeptical varies from consumer to consumer.

The cognitive approach provides an explanation for consumer skepticism of persuasive communication (Hovland et al., 1953). Within this approach, the persuasion knowledge model (Friestad and Wright, 1994) states that consumers learn to interpret and evaluate the persuasion agents' goals and tactics and use this knowledge to cope with persuasion attempts. Consumers use the resulting knowledge to identify situations that motivate skepticism. Research on skepticism has been developed in different contexts, such as corporate social marketing (Forehand and Grier, 2003), environmental claims (Mohr et al., 1998), communication of CSR (Vanhamme and Grobben, 2009), and CSR programs (Pirsch et al., 2007). As a result, communicating CSR initiatives may be problematic (Pomering and Dolnicar, 2009) because consumer frequently perceive these initiatives as marketing actions that companies engage in out of their own self-interests (Haniffa and Cooke, 2005). Therefore, inferred motivations determine the level of consumer skepticism toward CSR messages and the credibility of social actions. If consumers perceive a company's motivation as selfish, they will be more skeptical about the campaign and will give less credibility to the company communication activities (Groza et al., 2011). Because prior research has established that CSR campaigns include tactics that can raise suspicion of firm motives (Ellen et al., 2006), consumer skepticism can bias the perception of CSR engagement (Groza et al., 2011). Suspicion about CSR activities will be stronger for skeptical consumers than for non-skeptical consumers, with the subsequent negative impact on CSR associations. Thus:

*H4:* The more skeptical consumers are, the lesser their CSR associations will be.

### The relationship between CSR associations and attitudes

What consumers know about a company can influence their overall evaluations of and attitudes toward it (Luo and Bhattacharya, 2006). As part of their knowledge about the firm, consumers' perceptions of CSR are likely to influence their attitudes toward the firm and its social initiatives (Brown and Dacin, 1997). Attribution theory provides an appropriate framework for explaining how people attribute causes to events and how this cognitive perception affects their subsequent attitudes and behavior (Kelley and Michela, 1980). In this sense, CSR associations play an important role in consumers' responses to the company because they create a general context for evaluations (Sen and Bhattacharya, 2001). Consumers evaluate companies as well as their products in terms of CSR, and their perceptions of the motives for engaging in CSR influence their evaluations of and responsiveness to CSR (Becker-Olsen et al., 2006; Ellen et al., 2006).

Prior research has shown that consumers who are aware of a CSR initiative view the company as socially responsible (Brown and Dacin, 1997; Bhattacharya and Sen, 2004). As a result, the CSR activity has the potential to increase CSR associations and attitudes (Sen et al., 2006). If consumers believe that a company is concerned with the well-being of society and is committed to “doing good,” they are more likely to have favorable attitudes toward the company (Stanaland et al., 2011). More specifically, consumers who are aware of CSR initiatives report more positive attitudes and behavioral intentions (Öberseder et al., 2013). Accordingly, we expect that these positive associations with CSR actions lead to more positive attitudes toward the company.

*H5:* CSR associations have a positive direct effect on consumers' attitudes toward the company.

**Figure 1:**
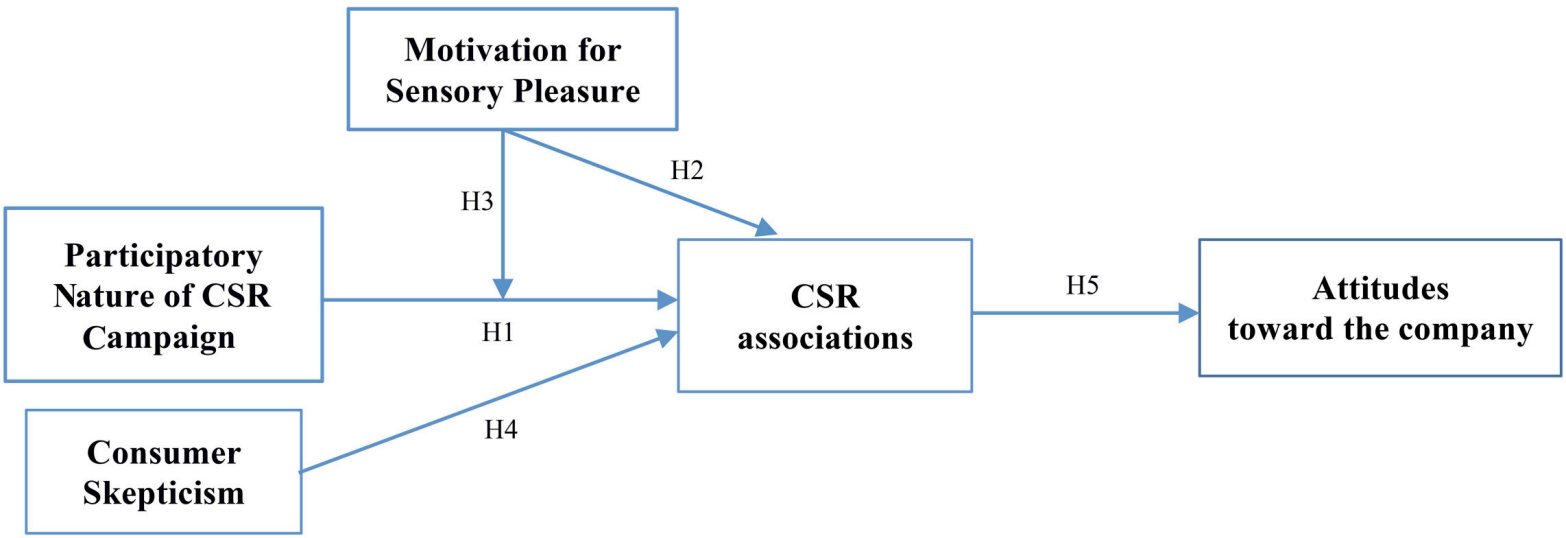
**Conceptual model**.

Figure 1 illustrates our conceptual model.

## Method

### Sample and procedure

We ran a field study in which 196 people were randomly selected on the street of a medium- sized European city. Upon arrival at the lab, participants were randomly assigned to one of two experimental conditions: (1) a participatory CSR campaign condition or (2) a non-participatory CSR campaign condition. They were exposed to an advertisement of a fictitious CSR campaign developed by a local brewer. The campaign was related to reforestation. In the participatory condition, the participants were invited to take part in the campaign by planting a tree, while the non-participatory scenario was purely informative and indicated that the company performed reforestation activities. After that, participants reported their attitudes toward the company, CSR associations, search for sensory pleasure, and skepticism. Participants took approximately 10 min to complete the questionnaire.

The sample included 94 men (47.96%) and 102 women (52.04%), ranging from 18 to 35 years of age (*M* = 24.35), with 66.33% between 18 and 25 years. Graduate respondents accounted for 6.12%, undergraduates represented 30.10%, and respondents with a high school education (61.73%) or less (2.04%) accounted for the remainder. All research activities were performed in accordance with University of Murcia's institutional review board policies concerning research with human subjects. Prior to participation in the study, we gave participants an information sheet and told them the activity was part of an experiment. After completing the questionnaire, they were thanked and debriefed.

The questionnaire comprised four scales adapted from previous research. We used three seven-point semantic differential scale items adapted from Lafferty and Goldsmith (2005) to measure attitude. We assessed CSR associations with a four-item Likert scale adapted from Dean (2002). We measured motivation for sensory pleasure with five seven-point scale items (1 = strongly disagree; 7 = strongly agree) adapted from Eisenberger et al. (2010). Finally, we used Skarmeas and Leonidou's (2013) six seven-point semantic differential scale items for skepticism and two items for the manipulation check (did company X ask for your participation in the reforestation campaign? yes/no; did the campaign ask you to do something specific? yes/no).

### Measurement assessment

Preliminary versions of the questionnaire were administered to a convenience sample of 20 consumers. We used the pretest results to improve the measures and design an appropriate structure for the questionnaire. Regarding the manipulation check, the 98 participants exposed to the participatory campaign confirmed that the campaign was participatory as required, while the 98 participants exposed to the non-participatory campaign confirmed the opposite.

We performed a validation check for the resulting measurement scales to assess their reliability, validity, and unidimensionality. We evaluated the reliability of the constructs using Cronbach's alpha coefficients (see Table 1). Cronbach alphas for the four constructs were above 0.70.

**Table 1:** **Construct and measures**.

**Item**	**^*y*^**	***t***	**mean**	**s.d**.	**ρ_c_**	**AVE**	**α**
*Attitude toward the company*					0.96	0.88	0.96
1. Positive/negative	0.94	NA	5.78	1.15			
2. Favorable/unfavorable	0.94	11.05	5.79	1.10			
3. Good/bad	0.94	14.70	5.77	1.22			
*CSR associations*					0.91	0.71	0.90
1. The company performs positive activities for society	0.88	NA	5.30	1.20			
2. The company does something for society	0.92	15.42	5.22	1.27			
3. The company cares about the welfare of its customers	0.68	11.67	4.88	1.29			
4. The company is socially responsible	0.87	16.55	5.23	1.38			
*Motivation for sensory pleasure*					0.89	0.42	0.87
1. I usually go to see nice landscapes	0.66	NA	4.66	1.62			
2. I enjoy the smell of nature	0.91	10.08	4.19	1.93			
3. I like stopping to feel nature	0.94	10.00	3.76	1.80			
4. I like walking in natural spaces	0.63	10.36	5.10	1.54			
*Skepticism*					0.77	0.62	0.87
1. The company tries to improve the welfare of society	0.81	NA	5.19	1.21			
2. The company follows high environmental standards	0.76	9.39	4.86	1.20			

Confirmatory factor analysis tested the measurement model and obtained acceptable overall model fit statistics [$\chi_{\left(59\right)}^{2}=118.08$, *p* ≈ 0.00; RMSEA = 0.07; SRMR = 0.06; NNFI = 0.95; CFI = 0.96]. We assessed reliability using the composite reliability index and the average variance extracted (AVE) index. For all the measures, both indices were higher than the evaluation criteria of 0.60 and 0.50, respectively (Bagozzi and Yi, 1988), as Table 1 shows. In line with Fornell and Larcker's (1981) suggested procedures, the scales showed acceptable convergent and discriminant validity. We assessed convergent validity by verifying that all indicators had statistically significant loadings on their respective latent constructs. The robust standard errors resulting from the use of the asymptotic covariance matrix were substantially larger (and the *t*-values smaller) than those produced by a model using the standard covariance matrix as input, validating the need for revised structural equation modeling (SEM) procedures in the face of strong non-normality in the data set. We also have evidence of discriminant validity. First, the phi matrix and associated robust standard errors presented in Table 2 ensured that unit correlation among latent variables was extremely unlikely (Bagozzi and Yi, 1988). Second, for all the pairwise relationships in the phi matrix, the AVE for each latent variable exceeded the square of the correlation between the variables.

**Table 2:** **Phi matrix of latent constructs for full sample**.

	**Attitude toward the company**	**CSR associations**	**Motivation for sensory pleasure**	**Skepticism**
Attitude toward the company	1.00 (0.17)			
CSR associations	0.30 (0.10)	1.00 (0.18)		
Motivation for sensory pleasure	0.14 (0.09)	0.02 (0.09)	1.00 (0.22)	
Skepticism	0.41 (0.11)	0.61 (0.13)	0.20 (0.10)	1.00 (0.21)

Standard errors in parentheses.

To provide a further check of discriminant validity, for each pair of the latent variables, we compared the scaled difference chi-square statistic of the hypothesized measurement model with a second model that constrained the correlation between those two latent variables to unity. The corrected chi-square difference tests using the Satorra–Bentler scaled chi-square values (Satorra and Bentler, 2001) indicated that the hypothesized measurement model was always superior to the constrained models. As a result, we are confident that each of the latent variables in our model exhibits discriminant validity with all other latent variables. Internal consistency and discriminant validity results enabled us to proceed with the estimation of the structural model.

Assessment of potential common method bias was analyzed following the recommendations of Lindell and Whitney (2001). We used the smallest positive value within the correlation matrix as a conservative estimate of bias. This happens to be the correlation between the motivation for sensory pleasure and CSR associations (*r* = 0.02). When we determined the statistical significance of the adjusted correlations, none of the correlations which were significant before the adjustment lost significance after the adjustment, indicating that the hypothesized relationships were not impacted by CMV.

## Results

Table 3 reports the results of the SEM applied to test the hypotheses proposed in the theoretical model. We again used the asymptotic covariance matrix and robust maximum likelihood in model estimation. The model fit the data acceptably, as evidenced by the goodness-of-fit measures [$\chi_{\left(119\right)}^{2}=228.98$, *p* = 0.00; RMSEA = 0.069; SRMR = 0.077; NNFI = 0.95; CFI = 0.96].

**Table 3:** **Model testing**.

**Relationships supported**	**Hypotheses**	**β (Standard error)**
Participatory nature of CSR campaign–CSR associations	H1	0.13 (0.153)
Motivation for sensory pleasure–CSR associations	H2	−0.35 (0.13)^***^
Participatory nature of CSR campaign × Motivation for sensory pleasure–CSR associations	H3	0.41 (0.15)^***^
Skepticism–CSR associations	H4	0.37 (0.09)^***^
CSR associations–Attitude toward the company	H5	0.31 (0.07)^***^

*** p < 0.001.

We tested the effect of the participatory nature of the CSR campaign on CSR associations. While the main effect of the participatory nature of the CSR campaign did not significantly influence CSR associations (β = 0.13; *SE* = 0.15), thus rejecting H1, its interaction with sensory pleasure did (β = 0.41; *SE* = 0.15), in support of H3. Therefore, on the basis of these results, we can affirm that sensory pleasure seeking moderates the effects of the participatory nature of the CSR campaign on CSR associations. The negative coefficient of the main effect of sensory pleasure seeking (β = −0.35; *SE* = 0.13) confirms the direct effect of sensory pleasure, as proposed in H2. In addition, in H4 we predicted that the more skeptical the consumers, the lesser their CSR associations would be, and our statistical test also found support for this relationship. That is, less skeptical consumers have greater CSR associations (β = 0.37; *SE* = 0.07). Accordingly, if consumers are less suspicious about the real motives of a CSR campaigns, their associations with CSR actions are more positive.

Finally, the results confirm that CSR associations exert a significant and positive influence on consumers' attitudes toward the company (β = 0.31; *SE* = 0.07), as predicted in H5. Thus, as consumers generate more positive CSR associations, their attitudes toward the company become more favorable.

The significant interaction effect was further analyzed through floodlight analysis using Johnson-Neyman's approach (Spiller et al., 2013), in order to calculate the range of values of sensory pleasure seeking for which the participatory nature of the CSR campaign has an effect on CSR associations different from zero. Results, obtained with the probemod R package (Tang, 2015), show that the effect of the participatory nature of the CSR campaign on CSR associations is positive and significant (i.e., confidence interval does not contain zero at *p* = 0.01) for values of sensory pleasure seeking above 5.02. With this information, we divide the sample into two groups, low sensory pleasure seeking subjects (with scores in this variable below 5.02) and high sensory pleasure seeking subjects (with scores above 5.02), with subsample sizes of 133 and 63, respectively.

While the variable sensory pleasure seeking has been hypothesized in our study to interact only with the participatory nature of the CSR campaign, for the multi-group analysis we also considere its potential effect on the other relationships, as a way to check whether these interactions should have been included in the original model. Therefore, a model that imposed equality constraints on the three parameters (participatory nature of the CSR campaign—CSR associations, skepticism—CSR associations, and CSR associations—attitude toward the company) and a general model that allowed those parameters to vary freely across subgroups were compared. A chi-square difference test revealed that the unconstrained model represented a significant improvement in fit over the constrained model (Δχ2 = 14.60; ΔDF = 3, *p* < 0.01). This result provide initial evidence to support the moderating effect of sensory pleasure seeking on the structural model.

A further series of tests identified there is only one path moderated by sensory pleasure seeking (Table 4). More specifically, the results showed a significant moderating effect consistent with H3. For high sensory pleasure seekers, being exposed to a participatory CSR campaign (compared to a non-participatory one) has a significant influence on CSR associations (β = 1.10; *SE* = 0.24). However, this effect is not significant for low sensory pleasure seekers (β = −0.23; *SE* = 0.19). The significant change in chi-square (Δχ2 = 7.49; ΔDF = 1, *p* < 0.01) indicates that this coefficient is different for the two groups. Additionally, the coefficients for the other two relationships displayed in Table 4 are significant for the two groups and the changes in chi-square indicate that they are not significantly different between the two groups. In other words, seeking sensory pleasure does not moderate the effect of skepticism on CSR associations nor the effect of the latter variable on consumer attitudes toward the company. In summary, these results fully confirm H3.

**Table 4:** **Model testing for the multi-group analysis**.

**Relationship**	**High motivation for sensory pleasure (*N* = 133)**	**Low motivation for sensory pleasure (*N* = 63)**	**Δχ2 (ΔDF = 1)**
Participatory nature of CSR campaign–CSR associations	1.10^***^	−0.23	7.49^***^
Skepticism–CSR associations	0.24^**^	0.45^***^	0.08
CSR associations–Attitude toward the company	0.23^**^	0.35^***^	1.79

*** p < 0.01;

** p < 0.05.

The negative effect of seeking sensory pleasure on CSR associations can be related to how the company CSR activities have been described. As Eisenberger et al. (2010) demonstrate, high motivation for sensory pleasure individuals show increased interest in high- but no low-detail contextual information about the pleasantness character of the campaign. In other words, these subjects preference for very detailed information about possibilities of experiencing pleasure could have provoked a sense of frustration and lack of interest in the experiment stimuli as they were not very detailed when describing the company CSR activities (but this is a common characteristic to many ads). This lack of interest may have favor lower processing of the information and, therefore, less CSR associations.

We ran an additional study to provide further support to the scenario we used. All research activities were performed in accordance with University of Murcia's institutional review board policies concerning human subjects research. We collected 41 questionnaires. Twenty-one participants were assigned to the participatory campaign whereas the remaining 20 were assigned to the non-participatory campaign. Through items ranging from 0 to 10, individuals rated the campaign in terms of credibility, realism, level of participation required and level of involvement required. They also rated their ability to imagine themselves immersed in the situation described. Results showed that both groups perceived the scenario they were exposed to as highly credible [*M*_np_ = 7, 15; *M*_p_ = 7, 62; *F*_(1, 39)_ = 1.43; *p* > 0.10] and realistic [*M*_np_ = 7, 40; *M*_p_ = 7, 86; *F*_(1, 39)_ = 1.63; *p* > 0.10], regardless of the type of campaign. They were also able to imagine themselves in the scenario [*M*_np_ = 7, 35; *M*_p_ = 7, 95; *F*_(1, 39)_ = 2.03; *p* > 0.10]. As expected, there were significant differences between the two conditions in terms of level of participation [*M*_np_ = 4, 10; *M*_p_ = 7, 05; *F*_(1, 39)_ = 20.57; *p* < 0.01] and involvement [*M*_np_ = 4, 60; *M*_p_ = 7, 62; *F*_(1, 39)_ = 20.26; *p* < 0.05], with higher scores for those in the participatory condition, which lends further support to our methodology. In summary, despite our manipulation was based on scenarios instead of real participation, subjects immersed themselves in those scenarios and those assigned to the participative condition indicated they perceive the CSR campaign as more participative than participants in the regular CSR campaign.

## General discussion

In the current competitive marketplace, companies intensely seek differentiation and credibility. One mechanism to reach such goals is consumer participation in CSR campaigns. However, while the effect of participation on consumer behavior has received considerable attention (Dellande et al., 2004; Chan et al., 2010), whether consumer participation in CSR activities increases the effectiveness of these activities remains unknown. The current research lends support to the contention that by proactively engaging consumers in CSR initiatives, firms can generate more favorable attitudinal responses than by acting in a reactionary manner (Becker-Olsen et al., 2006; Wagner et al., 2009).

Our findings on the effect of the participatory nature of a CSR campaign constitute a significant contribution both to the theory of consumer behavior and to business management. Thus, from a theoretical perspective, this research contributes to a better understanding of the effects of the participatory nature of CSR campaigns on consumer behavior. Specifically, although we did not find a general effect of the participatory nature of the campaign on consumers' perceptions of CSR activities, this does not mean that this effect does not exist. As the interaction shows, this effect is associated to consumers who are highly motivated to seek sensory pleasure. When the campaign is participatory, sensory pleasure seekers have greater perceptions of CSR activities than when the campaign is non-participatory. However, when consumers do not seek sensory pleasure, the fact that the campaign can offer possibilities to interact with the senses does not contribute to increase their CSR associations concerning the company. These results are in line with prior research suggesting that the motivation for sensory pleasure plays an important role in consumer responses (Eisenberger et al., 2010; Nurse et al., 2010) as well as those suggesting that enabling consumers participation leads to more positive outcomes (Chan et al., 2010; Yi et al., 2011; Troye and Supphellen, 2012; Mustak et al., 2013; Olsen and Mai, 2013; Atakan et al., 2014). In summary, our results show that motivation and involvement positively moderate the effect of a message on the valence of consumes' associations. A positive message (CSR activities undertaken by the company) contributes to more positive associations (CSR associations) when the consumer is more motivated (seek sensations) and involved (participates in the production of the CSR activity).

In addition, in light of the negative attributions consumers may attach to companies' real motives for conducting CSR actions, our research demonstrates that skeptical consumers show less CSR associations because they may perceive the campaign as manipulative. This, in turn, results in less favorable attitudes toward the company. This result is in line with research that posits that consumers are often skeptical of advertising claims related to a company's participation in social or environmental issues (Obermiller and Spangenberg, 1998; Chan et al., 2010).

From a managerial perspective, this research shows that, by itself, participation may not be enough to gain consumers' involvement and, in turn, generate greater CSR associations and favorable attitudes. To obtain this effect, companies should emphasize the possibility of finding pleasure during the CSR campaign. Firms could try to activate sensory pleasure, beyond the personal predisposition of each individual, by promoting CSR as an action that will produce an enjoyable experience as involvement increases. Therefore, marketing managers should not only engage their customers in their CSR actions, so that they actively take part in their implementation (as opposed to adopting a passive role and trusting that the company will do what is promised), but also design participatory CSR activities that stimulate and match the consumers' hedonic motivations for searching for pleasure.

In addition to participation and sensory pleasure seeking, marketers should pay attention to skepticism of the campaign. Some consumers tend to discredit CSR actions, as they interpret them as an attempt to manipulate their perceptions. They believe that the real motives behind CSR actions are not social oriented but benefit oriented. To minimize the impact of such skepticism, managers should provide consumers with clues that lend the campaigns more credibility. For example, they could report the results of previous initiatives to prove that the company is socially oriented and concerned about societal well-being.

Despite the findings and implications, this research has some limitations. First, although participants were able to imagine themselves in the proposed scenarios, we must acknowledge that they do not allow consumers to really participate in the CSR campaign. Therefore, further research should assess whether our findings remain the same in such a real context. Second, we use only one company, which limits the generalizability of the results. Thus, future research should analyze whether the implementation of participatory CSR activities for different products can generate different results for different company sectors. Third, other variables related to consumer–company interactions, such as the relationship with company workers, may also affect the results. Finally, another avenue for research pertains to consumers' personality traits, which may moderate the effects. For example, proenvironmental attitudes or prosocial behavior may boost the positive influence of participation.

## Author contributions

RL collected the data and the three authors have equally participated in literature review, data analysis and writing of the paper.

## Funding

The author(s) disclosed receipt of the following financial support for the research, authorship, and/or publication of this article: This research was supported by the grant ECO2012-35766 from the Spanish Ministry of Economics and Competitiveness and by the Fundación Séneca-Agencia de Ciencia y Tecnología de la Región de Murcia (Spain), under the II PCTRM 2007-2010. Authors also thank the support provided by Fundación Cajamurcia.

### Conflict of interest statement

The authors declare that the research was conducted in the absence of any commercial or financial relationships that could be construed as a potential conflict of interest.
